# Comprehensive comparison of antioxidant properties of tinctures

**DOI:** 10.1038/s41598-019-42656-2

**Published:** 2019-04-16

**Authors:** Justyna Polak, Mariola Bartoszek, Roksana Bernat

**Affiliations:** Department of Materials Chemistry and Chemical Technology, Institute of Chemistry, The University of Silesia, 9 Szkolna Street, 40-006 Katowice, Poland

## Abstract

Homemade tinctures, traditional Polish alcoholic beverages called “nalewkas” (similar to alcohol herbal tinctures), which antioxidant capacity have never been studied before, were characterized by electron paramagnetic resonance (EPR), nuclear magnetic resonance (NMR) and ultraviolet visible (UV-vis) spectroscopy. The antioxidant properties of nalewkas made according to homemade recipes were compared to commercially produced nalewkas. The impact of aging on antioxidant properties of nalewkas was investigated. The results showed that all of examined nalewkas exhibited strong antioxidant properties (antioxidant capacity TEAC_DPPH_ 466 μmol TE/100 mL – 11890 μmol TE/100 mL). It was found that the value of the antioxidant capacity corresponds to the total phenolic and aromatic proton content. The impact of the production method and the type of fruit used on the TEAC_DPPH_ value was also noted. The unripe walnuts with green husks has the highest value of the antioxidant capacity TEAC_DPPH_ (11890 µM/100 mL) not only for alcoholic beverages, but also among food products.

## Introduction

Fruits and fruit preserves are a valuable source of compounds with antioxidant properties, as well as some vitamins and minerals^1^. In literature, one can find numerous studies on the antioxidant properties of fresh fruits and vegetables, as well as products made from them, such as juices, purees, compotes. Some studies investigated antioxidant capacity (AC) of wines and the influence of parameters such as polyphenols, the origin of the fruit they were made of, alcohol and sugar content on AC values^2,3^. However, there are few reports in available literature on the antioxidant properties of nalewkas that is, alcoholic solutions containing polyphenol compounds from fruit as well as sugar^4–9^.

The term “nalewkas” doesn’t exist in Western Europe. One can encounter the terms: spirit drinks, spirit tincture preceded by the name of the fruit, or the term liquor (liqueur or cordial). It should be noted that liquor drinks are not the same as liqueurs. Liqueurs are spirit drinks with a high sugar content, while a tincture can be either sweet or savory. Liqueurs can also often be found under their own names, for example. Nocino – walnut tincture^4,5^, limoncello - lemon tincture^10^ Prunelle, Pacharan – sloeberry tincture^11^. Only a few research groups in Europe have conducted research on the antioxidant properties of the alcoholic beverages called nalewkas, tincture^4–9^.

The studies considered, among others, Polish commercial tinctures from the various fruit^7^. In addition, research was conducted on the composition and antioxidant properties of liqueurs obtained from walnuts (nocino), and myrtle fruits^4–6,9^. Moreover, research was also conducted on nalewkas made from rose flowers, Japanese apricots, and cherries, and on the aromatics of lemon nalewkas^12–17^. The biologically active ingredients in such alcoholic beverages, and therefore the ingredients responsible for their very good antioxidant properties, are considered to be similar to those in wines, and include, inter alia, flavonols (quercetin), resveratrol, proanthocyanidin, catechin and ferulic acid^18,19^.

In Poland, alcoholic tinctures called nalewkas are very popular. They are produced on the basis of herbs, roots and fruits. In Poland it was customary to prepare tinctures from the fruit grown in home gardens and orchards, from wild-growing fruit trees and shrubs as well as from wild herbs, flowers and roots. They were appreciated for their taste, color and aroma. Fruit, herbal and root nalewkas were also used in the treatment of various indispositions, utilizing the therapeutic effects of the substances included in their composition^7^. Homemade nalewkas are often stored for a longer period of time in basements (at low temperature and without sun exposition). A significant effect of aging of alcoholic beverages on antioxidant properties has been proved in many studies^20,21^.

Hence, the theme of this work is a study of the antioxidant capacity of Nalewkas produced according to traditional home recipes from fruit: raspberry, walnut, elderberry, cowberry, bilberry, blackberry, aronia, cranberry, rowanberry, cherry as well as mixed nalewkas. The impact of aging on antioxidant capacity was also investigated. To determine the TEAC_DPPH_ antioxidant capacity, EPR spectroscopy was applied. The resulting values of TEAC_DPPH_ antioxidant capacity were correlated with the total phenolic compounds obtained by the FC method and with aromatic proton content by NMR spectroscopy. In addition, the antioxidant properties of nalewkas made according to home recipes were compared to commercial nalewkas.

## Materials and Methods

### Chemicals and nalewkas

DPPH^•^(Sigma-Aldrich, Poznań, Poland) was used as the source of free radicals. To quantify the antioxidant capacity of the tinctures, trolox (Acros Organics, Geel, Belgium) was used. In order to determine total polyphenol, the FC reagent and gallic acid (POCH, Gliwice, Poland) were used. All other chemicals and solvents were of analytical grade and used without further purification.

Fourteen homemade nalewkas (tinctures) differing in the main macerated ingredient, color, alcohol content, turbidity and pH, were included in the study (Table 1).

**Table 1:** Names of the samples, characteristics and TEAC, TP, H_Ar_, CI, turbidity and pH values obtained for the studied nalewkas.

Sample	Fruit	Latin name	TEAC_DPPH_ [µM/100 mL]	TP [mg GA/L]	H_Ar_ [%]	CI	Turbidity [NTU]	pH
S1	berry mixture	—	501 ± 18	1283 ± 26	0.009	7.68 ± 1.12	18 ± 3	3.64 ± 1.08
S2	dried fruits mixture	—	625 ± 218	994 ± 12	0.011	4.34 ± 0.87	72 ± 6	4.29 ± 0.87
S3	mint, lemon balm, lemon	—	1426 ± 19	860 ± 21	0.048	0.87 ± 0.11	11 ± 1	3.78 ± 0.45
S4	green walnut	*Juglans regia*	11890 ± 272	10302 ± 55	0.523	4.73 ± 0.09	19 ± 8	3.88 ± 0.32
S5	raspberry	*Rubus idaeus*	880 ± 15	792 ± 9	0.034	2.64 ± 0.08	24 ± 2	3.33 ± 0.08
S6	elderberry	*Sambucus nigra*	973 ± 32	802 ± 21	0.063	8.48 ± 1.11	18 ± 3	4.18 ± 0.53
S7	cowberry	*Vaccinium vitis-idaea*	788 ± 9	1235 ± 4	0.040	6.33 ± 0.50	225 ± 15	2.82 ± 0.09
S8	bilberry	*Vaccinium myrtillus*	1488 ± 24	1571 ± 45	0.063	13.93 ± 2.62	230 ± 34	3.10 ± 0.75
S9	blackberry	*Rubus fruticosus*	1374 ± 33	1244 ± 28	0.057	11.57 ± 1.98	655 ± 51	3.37 ± 0.89
S10	chokeberries	*Aronia melanocarpa*	2244 ± 9	3148 ± 21	0.072	10.80 ± 1.87	30 ± 4	3.44 ± 0.39
S11	cranberry	*Vaccinium oxycoccos*	466 ± 19	715 ± 11	0.008	4.42 ± 0.98	17 ± 2	2.66 ± 0.68
S12	green walnut	*Juglans regia*	5988 ± 3	3936 ± 34	0.125	8.81 ± 1.76	33 ± 8	3.72 ± 0.06
S13	rowanberry	*Sorbus aucuparia*	1366 ± 64	1302 ± 14	0.066	2.31 ± 0.79	25 ± 3	3.62 ± 0.25
S14	cherry	*Prunus cerasus*	1359 ± 26	3936 ± 19	0.092	10.83 ± 2.04	29 ± 9	3.67 ± 0.55

Abbreviations: TEAC_DPPH_: Trolox equivalent antioxidant capacity, TP: total phenolic, H_Ar_: aromatic protons content, CI: Color Intensity (420 + 520 + 620) Each value of TEAC and TP is presented as mean ± SD (n = 3).

Sample no. S1- homemade berries nalewka: mix of fruit (1 kg strawberries, 0.5 kg bilberries, 0.25 kg cowberries, 0.5 kg American blueberries, 0.5 kg cranberries, 0.5 kg chokeberry, 0.2 kg dried goji berries) and 0.1 kg white grain sugar and 0.1 kg cane were completely covered with 1.5 L 96% v/v ethyl alcohol; the mixture was shaken every seven days.

Sample no. S2 – homemade dried fruit nalewka: mix of dried fruits (0.15 kg dried plums, 0.15 kg dates, 0.15 kg figs, 0.15 kg apricots, 0.15 kg dried apples), 0.15 kg walnuts, 0.5 kg oranges with skin (previously blanched) dram of cinnamon, cloves, pepper, 0.2 kg sugar mixed together, covered with 0.7 L 96% v/v ethyl alcohol; the mixture was shaken every seven days.

Sample no. S3 – homemade herbal-fruit nalewka: 0.15 kg mint, 0.15 kg lemon balm, 0.5 kg honey, 0.1 kg xylitol, 0.5 kg lemon (previously blanched, with peel) were mixed with 0.5 L 40% v/v ethyl alcohol; the mixture was shaken every seven days.

Sample no. S4 – homemade green walnuts nalewka: 2 kg unripe walnuts with green husks were washed in hot water, inserted into a bottle and mixed with 0.5 L 40% v/v ethyl alcohol; the mixture was shaken every seven days.

Sample no. S5- homemade raspberries nalewka: 1 kg raspberries and 0.4 kg sugar were added into a jar. The contents of the jar were mixed from time to time for 3–4 days; afterwards alcohol (0.5 L 60% v/v ethyl alcohol) was added to the mixture. Next, the mixture was shaken every seven days.

Sample no. S6 – homemade elderberries nalewka: ripe elderberries (0.7 kg) were washed, mixed with 0.5 L water and boiled for about 5 minutes. The elderberries were filtered out and alcohol was added to the juice (0.7 L 40% v/v ethyl alcohol and 0.2 L 96% v/v ethyl alcohol). Lemon peel and lemon juice (0.5 kg lemon) were also added to the mixture. Next, the mixture was shaken every seven days.

Sample no. S7 – homemade cowberries nalewka: 0.5 kg cowberries, 0.25 kg white sugar, 0.25 L water and 0.25 L 96% v/v ethyl alcohol were mixed. Next, the mixture was shaken every seven days.

Sample no. S8 – homemade bilberries nalewka: 0.5 kg bilberry, 0.25 kg white sugar, 0.25 L water and 0.25 L 96% v/v ethyl alcohol were mixed. Next, the mixture was shaken every seven days.

Sample no. S9 – homemade blackberries nalewka: 0.5 kg blackberries, 0.25 kg white sugar, 0.25 L water and 0.25 L 96% v/v ethyl alcohol were mixed. Next, the mixture was shaken every seven days.

Sample no. S10 – homemade chokeberries nalewka: 0.5 kg aronia 0.25 kg white sugar, 0.25 L water and 0.25 L 96% v/v ethyl alcohol were mixed. Next, the mixture was shaken every seven days.

Sample no. S11 homemade cranberries nalewka: 0.5 kg cranberries, 0.25 kg white sugar, 0.25 L water and 0.25 L 96% v/v ethyl alcohol were mixed. Next, the mixture was shaken every seven days.

Sample no. S12 homemade green walnuts nalewka: 0.5 kg unripe walnuts with green husks, 0.25 kg white sugar, 0.25 L water and 0.25 L 96% v/v ethyl alcohol were mixed. Next, the mixture was shaken every seven days.

Sample no. S13 – homemade rowanberries nalewka: 0.5 kg rowanberry, 0.25 kg white sugar, 0.25 L water and 0.25 L 96% v/v ethyl alcohol were mixed. Next, the mixture was shaken every seven days.

Sample no. S14 – homemade cherries nalewka: 2 kg pitted cherries and 1.5 kg sugar were covered with 1 L 96% v/v ethyl alcohol. Next, the mixture was shaken every seven days.

All samples were macerated in closed containers for one month. Afterwards nalewkas were decanted, poured into bottles and stored in the dark. In case of walnut nalewka (sample no. S4), walnut green husks remained in the bottle without decanting.

### Determination of antioxidant capacity by EPR spectroscopy

Antioxidant capacity (TEAC_DPPH_) was determined using the method described previously^7,22^.

A typical reaction mixture contained 1 mL of 200 µmol/L DPPH^•^ solution in ethanol together with appropriate volume of nalewka, depending on the manifested antioxidant properties. For all samples, the regression equation of the linear relationship between the percent inhibition (*%I*) of the EPR signal intensity and the volume of nalewka sample (*V*) was determined. Based on this equation the *%I* corresponding to 100 mL of the studied nalewka was calculated. Then, from the standard curve obtained for trolox: y = 987.60x + 16.36, where: y is the inhibition [%] and x is the volume of the sample [mL] the antioxidant capacity given in µmol TE per 100 mL of nalewka was defined^7,22^. The data presented here are the result of three trials.

EPR spectra were obtained with a Bruker EMX EPR spectrometer (Bruker-Biospin, Karlsruhe, Germany) operating at the X-band frequency at room temperature. The typical instrument parameters were: central field, 3480 G; modulation amplitude, 2.0 G; time constant, 40.96; gain, 1*10^4^ G; microwave power, 20.12 mW.

### Determination of total polyphenols content

The total polyphenol content in the nalewkas was measured by the Folin Ciocalteau spectrophotometric method described previously^2,7,19^.

### NMR Spectroscopy

^1^H NMR spectra were measured using the method described previously^3,7^.

### The impact of aging on antioxidant capacity of nalewkas

For 10 selected nalewkas (S4-S14) the impact of aging on their antioxidant properties was studied. Each nalewka was examined 6 times at appropriate intervals using EPR spectroscopy.

### Statistical analysis

The presented data are the mean values whereas errors were calculated as standard deviation (SD). One-way analysis of variance (ANOVA) was performed with a level of significance of p = 0.05. Correlation coefficients (r): TEAC_DPPH,_ TP, H_ar_, pH and turbidity were calculated by the Pearson test with a level of significance of p = 0.05. The statistical analyses were carried out using OriginPro 8.5.

### Compliance with Ethical Requirements

Ethical approval: This article does not contain any studies with human participants or animals performed by any of the authors.

## Results and Discussion

### The antioxidant capacity (TEAC_DPPH_) of homemade nalewkas

The antioxidant capacity of nalewkas was measured using the EPR spectroscopy. Based on the conducted research it can be concluded that all of the examined homemade nalewkas exhibited strong antioxidant properties (Table 1). The values of TEAC_DPPH_ determined by the EPR spectroscopy were within the range of 466 μmol TE/100 mL –11890 μmol TE/100 mL (Table 1).

Comparing the results obtained for homemade nalewkas with those for other alcoholic beverages such as wine or beer, known for their very good antioxidant properties, it can be stated that homemade nalewkas stand out in terms of the value of the antioxidant capacity. In case of wines the value of the antioxidant capacity amounts from 16 to 3780 μM TE/100 mL for beers the antioxidant capacity ranges from 75 to 333 μM TE/100 mL – these results were obtained only for beer with addition of natural cherry juice (TEAC_DPPH_ = 795 μM TE/100 mL)^23,24^. In case of hard liquors, with significantly lower antioxidant characteristics, the antioxidant capacity is even lower and ranges from 61 to 115 μM TE/100 mL for whisky, from 41 to 56 μM TE/100 mL for flavoured vodkas, whereas “clear” alcohols show no antioxidant capacity^22^. Comparing the antioxidant capacity of the examined nalewkas with that of other food products, like coffee, tea, juice, fruits or vegetables, nalewkas perform best of all^25–27^.

The highest value of the antioxidant capacity TEAC_DPPH_, significantly standing out amongst all other results, was obtained for nalewka prepared from walnuts – nalewka S4 (Table 1). This value – 11890 μmol TE/100 mL – is the highest value of the antioxidant capacity ever obtained for a food product. The lowest value of the antioxidant capacity TEAC_DPPH_ was obtained for nalewkas obtained from mix of berry S1 and cranberries S11 (Table 1). It should be noted though that the obtained values of the antioxidant capacity TEAC_DPPH_, although the lowest amongst the researched nalewkas, are still very high and characteristic rather for food products like wine, green tea, coffee^26,27^, characterised by very good antioxidant capacities. The differences between the antioxidant capacities of the individual nalewkas are the result of the antioxidant capacities of the fruit they were prepared from and the preparation method (recipe).

The highest value of the antioxidant capacities, as already mentioned above, has been obtained for both nalewkas made from unripe walnuts with green husks. The difference between these two nalewkas is very high: for nalewka S4 the TEAC_DPPH_ value is 11890 μM TE/100 mL, whereas for nalewka S12 the TEAC_DPPH_ value is 5988 μM TE/100 mL, so about half of the one for nalewka S4. Such a significant difference between the values of the antioxidant capacity is most probably caused by different preparation methods (recipes). In nalewka S4, the unripe walnuts with green husks are constantly submerged in alcohol, while in nalewka S12 the fruits were drained after one month of being covered with alcohol (Table 1). Probably due to the fact that the fruits remain submerged in alcohol, the compounds with antioxidant characteristics are gradually released, resulting in the increase of the antioxidant capacity of the researched nalewka. High values of antioxidant capacities of the walnut nalewkas are not a surprising result, as nuts are rich source of compounds with antioxidant properties; the following phenolic compounds were identified: hydrolysable taninins (mainly glansreginin A and B), chlorogenic acid, caffeic acid, ferulic acid, coumaric acid, gallic acid, ellagic acid, protocatechuic acid, syringic acid, vanillic acid, catechin (58–81µmol/g in raw nuts, 98–115 µmol/g in toasted nuts), epicatechin, procyanidine, myricetin, juglone, vitamin C (ascorbic acid) and vitamin E (tocopherol)^28,29^. Juglone is the major phenolic compound occurring in walnut green husks. A few studies reported that the total phenolic content in walnut liqueur made from unripe walnuts with green husk is higher than in liqueurs made from ripened fruits^4,5,28^.

Very good therapeutic characteristics of unripe walnuts with green husks nalewka are well known; it is a proven remedy for problems related to the human digestion system. It also shows beneficial effects against stomachaches, food poisoning and digestive issues^28^.

A high value of the antioxidant capacity TEAC_DPPH_ was also obtained for aronia nalewka S10–2244 μM TE/100 mL. Chokeberries are characterised by high antioxidant capacity and high polyphenol content. The total polyphenol content, according to the literature data, is quantified at 40–70 mg/g d.m., with as much as 50% of it being anthocyanins. Apart from anthocyanins, an important group of compounds contained in chokeberries are the hydroxycinnamic acid derivatives, which together with flavanols are responsible for the tart flavour of chokeberries and very high antioxidant capacity^30,31^.

Next, in terms of the antioxidant capacity, is bilberry nalewka S8 (TEAC_DPPH_ = 1488 μM TE/100 mL) and herbal-fruit nalewka S3 (TEAC_DPPH_ = 1426 μM TE/100 mL). Good antioxidant characteristics of bilberries result from the presence of anthocyanins and *p*-terostilben, which reduce the antioxidant stress related to the presence of reactive oxygen species.

Nalewkas prepared from cherries S14 (TEAC_DPPH_ = 1359 μM TE/100 mL), rowanberries S13 (TEAC = 1366.68 μM TE/100 mL) and blackberries S9 (TEAC_DPPH_ = 1374 μM TE/100 mL) are characterised by almost identical values of the antioxidant capacity. Good antioxidant capacity of cherry nalewka results from the presence of provitamin A, vitamin C, hydroxycinnamic acids (chlorogenic acid) (180–1150 mg/kg fresh mass), anthocyanidins (malvidin) (350–4500 mg/kg fresh mass), anthocyanins (cyanidin glycosides) (do 60,6 mg/100 g), flavanols (epicatechin) (50–22 mg/kg fresh mass) and carotenoids in cherry fruits. Additionally, cherries are rich in copper, a microelement responsible for correct course of cellular respiration^32,33^. Rowanberries are a rich source of substances with antioxidant properties; they contain about 12 mg of carotenoids in 100 g of berries. Moreover they contain: hydroxybenzoic acids (protocatechuic acid and ellagic acid) (0.8–7 mg/100 g fresh mass), hydroxycinnamic acids (*p*-coumaric acid) (4–22 mg/100 g fresh mass), anthocyanins (peonidin glycosides and cyanidin glycosides) (3 mg/g dry mass, 30–86 mg/100 g fresh mass, and 121 mg/100 g purred fresh mass), flavonols (quercetin, myricetin) (23–27 mg/100 g fresh mass, 358 mg/100 g pureed fresh mass), flavanols (catechins and epicatechins) (1–412 mg/100 g fresh mass) and vitamin C^34,35^.

Blackberries in turn are characterised by high content of phenolic compounds (23 mg/g d.m. gallic acid equivalents). The blackberry flesh contains cyanidins glycosides and quercetin glycosides, whereas the blackberry seed contain significant amounts of ellagic acid, epicatechins and procyanidins^36^.

The antioxidant capacity below 1000 μM TE/100 mL characterises nalewkas made from elderberry S6 (TEAC_DPPH_ = 973 μM TE/100 mL), raspberry S5 (TEAC_DPPH_ = 880 μM TE/100 mL) and cowberry S7 (TEAC_DPPH_ = 788 μM TE/100 mL). The phenolic compounds are responsible for the antioxidant characteristics of the elderberry nalewka (20 mg/g d.m.), represented mainly by anthocyanins: cyanidin glycosides^37^. Garden raspberries contain from 15 till 30 mg/100 g of vitamin C, polyphenols, 50% of which are ellagic acid and anthocyanins^38^. Cowberries own their antioxidant characteristics mainly to the presence of quercetin^39^, anthocyanins, vitamin A, E and C, ellagic acid and folic acid. Moreover, the presence of such minerals as selenium, zinc, manganese and copper contributes to the increased effectiveness of the antioxidant properties.

The weakest antioxidant characteristics were observed in berry mix nalewka S1 (TEAC_DPPH_ = 501 μM TE/100 mL), dry fruit nalewka S2 (TEAC_DPPH_ = 625 μM TE/100 mL) and cranberry nalewka S11 with the lowest TEAC_DPPH_ value of 466 μM TE/100 mL. It was repeatedly proven that the antioxidant capacity of a mix of products with good antioxidant characteristics is lower than that of each one of them separately, e.g. mixed fruit juice containing blackcurrant or orange juice shows lower antioxidant capacity that the blackcurrant or orange juice on its own. Therefore, even though it would seems that berry nalewka would have very high antioxidant capacity due to each fruit’s very good antioxidant characteristics, the mix of berry performs worse. However it should be emphasized that nalewkas: S1, S2 and S11, despite the fact of having the weakest antioxidant characteristics of all homemade nalewkas, stand out in terms of very high values of the antioxidant capacity amongst alcoholic beverages and even amongst food products.

### The relationship between antioxidant capacity (TEAC_DPPH_) of nalewkas obtained according to homemade and commercial recipe

The research of commercially produced antioxidant nalewkas was previously performed^7^. These nalewkas were acquired in local stores and examined according to the same method. The standard for free radicals was DPPH, and the research was conducted using the EPR spectroscopy^7^.

For homemade nalewkas the TEAC_DPPH_ value ranges between 466 and 11890 μM TE/100 mL (Table 2), for commercially produced nalewkas this value is significantly lower and ranges between 45 and 1045 μM TE/100 mL (Table 2). While comparing commercially produced and homemade nalewkas made from the same fruits one can observe that in case of both examined commercially produced cranberry nalewkas, nalewka produced according to homemade recipe exhibit 10 times higher antioxidant capacity TEAC_DPPH_. The same tendency is observed for rowanberry homemade nalewka that shows over 20 times higher TEAC_DPPH_ value than the commercially produced equivalent (Table 2). Commercially produced cranberry and rowanberry nalewkas possess the ability to bind free radicals on the level characteristic rather for the flavoured vodkas (TEAC_DPPH_ = 41–56 μM TE/100 mL) than nalewkas (Table 2). Depending on the production recipe, walnut nalewka is characterised by as much as 90 times (for S4) or 45 times (for S11) higher TEAC_DPPH_ value than the commercially produced equivalent (Table 2). The literature data on the antioxidant capacity determined for the commercial Italian liqueurs from walnuts (nocino) are closer rather to the commercially produced nalewkas than the homemade ones^4,5^. In case of raspberry nalewkas, the TEAC_DPPH_ value for homemade nalewka is 8 times higher than the value obtained for commercially produced one, and TEAC_DPPH_ for homemade cherry nalewka is 10 times higher than the one of the commercially produced equivalent (Table 2). Two commercial cherry nalewkas from the same producer, differing in the production method and storage method were tested. Cherry nalewka (TEAC_DPPH_ = 133 μM TE/100 mL) was storage in oak barrels, contrary to the other commercial cherry nalewka (TEAC_DPPH_ = 126 μM TE/100 mL) yet it had no impact on the value of the antioxidant capacity^7^, as it was observed in case of whiskey^22^. Both nalewkas are characterised by comparable TEAC_DPPH_ values, what indicates that in that case the industrial production method and the way of storage do not influence the antioxidant potential in significant way.

**Table 2:** Antioxidant capacity of nalewkas obtained according to homemade and commercial recipe.

Fruit	Homemade nalewkas TEAC_DPPH_ [μM TE/100 mL]	Commercial nalewkas TEAC_DPPH_ [μM TE/100 mL] (Polak & Bartoszek, 2015)
Elderberry	973 ± 33	1045 ± 32
Cranberry	466 ± 19	45 ± 70
	—	48 ± 60
Green Walnut	11890 ± 270	132 ± 27
	5988 ± 30	—
Raspberry	880 ± 15	108 ± 14
Rowanberry	1367 ± 64	60 ± 16
Cherry	1359 ± 26	127 ± 90
	—	133 ± 20

Abbreviations: TEAC: Trolox equivalent antioxidant capacity.

In conclusion, homemade nalewkas exhibit notably better antioxidant characteristics than commercially produced nalewkas (Table 2). The only exception is the elderberry nalewka, where antioxidant characteristics of commercially produced nalewka are slightly better than a homemade one – the difference is at 7% (Table 2). Other homemade nalewkas are characterised by significantly higher values of the antioxidant capacity TEAC_DPPH_ than commercially produced ones.

The reason of such notable differences between the obtained results is the production method; nalewkas produced according to homemade recipes are obtained mainly through maceration – berries/fruits, sugar and pure alcohol are used in production, whereas commercial nalewkas are obtained through addition of aromas and artificial colouring. In addition, in case of commercially produced nalewkas, the product is subjected to clarification or filtration, which results in partial removal of biologically active antioxidants that are directly bound to the berry/fruit flesh.

### The relationship between TEAC_DPPH_ obtained by EPR spectroscopy, TP obtained by the FC method and H_ar_ obtained by NMR spectroscopy

The values of total phenolic content in the nalewka samples ranged from 715 to 10302 mg GA/L (Table 1). For the tested nalewkas, the high values of antioxidant capacity corresponded with the elevated content of total polyphenol. The analysis of the obtained TEAC_DPPH_ and TP values indicates a correlation between them (r = 0.94, p < 0.05).

An accurate assignment of individual signals to a particular polyphenols on ^1^H NMR spectra was not possible due to a low signal intensity in the aromatic proton region. In order to compare of the percentage of polyphenol content in the individual nalewkas the integration of the signals were performed (Table 1).

The total integrals of the NMR spectra for the aromatic region (6.2–8.2 ppm) obtained for the nalewkas correlate with the total phenolic content of the nalewkas (r = 0.97; p < 0.05) and TEAC_DPPH_ values (r = 0.96; p < 0.05). An analysis of the NMR spectra of the samples tested in this study confirms that majority of the studied nalewkas the highest antioxidant capacity was connected with the highest content of H_ar_. Correlation between TEAC_DPPH_ values and CI (r = −0.04; p < 0.05), Turbidity (r = −0.15; p < 0.05), pH (r = 0.25; p < 0.05) and correlation between TPC values and CI (r = 0.07; p < 0.05), Turbidity (r = −0.18; p < 0.05), pH (r = 0.24; p < 0.05) wasn’t observed.

### The impact of aging on antioxidant capacity of nalewkas

In order to determine the effect of aging on antioxidant properties and to determine the optimal aging time for nalewkas produced according to home recipes, the studies were performed where the antioxidant capacity of nalewkas was measured using the EPR spectroscopy method at different time intervals (Table 3).

**Table 3:** The impact of aging on antioxidant capacity of nalewkas.

No. of sample	Fruit	Date of production	Measurements	Date of measurement	Number of days	TEAC_DPPH_ [μM TE/100 mL]
S4	walnut	08.07.2015	1	23.10.2015	105	11890
			2	09.11.2015	121	13738
			3	04.12.2015	146	15434
			4	15.01.2016	187	16253
			5	29.01.2016	201	16140
			6	27.09.2016	439	12042
S5	raspberry	08.07.2015	1	23.10.2015	107	880
			2	09.11.2015	124	721
			3	04.12.2015	147	663
			4	15.01.2016	189	660
			5	22.01.2016	196	744
			6	27.09.2016	439	601
S6	elderberry	10.09.2015	1	16.10.2015	36	973
			2	30.10.2015	50	895
			3	19.11.2015	70	911
			4	11.12.2015	92	911
			5	29.01.2016	141	937
			6	27.09.2016	377	900
S7	cowberry	28.08.2015	1	23.10.2015	56	788
			2	09.11.2015	73	846
			3	04.12.2015	98	750
			4	15.01.2016	140	792
			5	29.01.2016	154	795
			6	28.09.2016	390	561
S8	bilberry	28.08.2015	1	23.10.2015	56	1488
			2	09.11.2015	73	2295
			3	04.12.2015	98	2240
			4	15.01.2016	140	2301
			5	29.01.2016	154	2251
			6	28.09.2016	390	2873
S9	blackberry	28.08.2015	1	23.10.2015	56	1374
			2	09.11.2015	73	1321
			3	04.12.2015	98	1371
			4	15.01.2016	140	1400
			5	29.01.2016	154	1429
			6	28.09.2016	390	1405
S10	chokeberry	28.08.2015	1	30.10.2015	63	2244
			2	19.11.2015	83	2044
			3	11.12.2015	105	2271
			4	22.01.2016	147	2119
			5	29.01.2016	154	1986
			6	27.09.2016	389	1842
S11	cranberry	09.09.2015	1	16.10.2015	37	466
			2	30.10.2015	51	526
			3	19.11.2015	71	466
			4	11.12.2015	93	674
			5	22.01.2016	135	620
			6	29.09.2016	380	498
S13	rowanberry	09.09.2015	1	16.10.2015	37	1367
			2	30.10.2015	51	1571
			3	19.11.2015	71	1561
			4	11.12.2015	93	1495
			5	22.01.2016	135	1548
			6	29.09.2016	380	1246
S14	cherry	14.09.2015	1	30.10.2015	46	1359
			2	19.11.2015	66	1987
			3	11.12.2015	88	2100
			4	22.01.2016	130	2089
			5	29.01.2016	137	2171
			6	29.09.2016	375	1723

Abbreviations: TEAC: Trolox equivalent antioxidant capacity.

Based on the conducted research, different changes in the antioxidant properties of nalewkas were observed during the aging process depending on the production method of the nalewka and type of fruit used. All observed differences were significant at 0.05 level.

The aging time had the greatest impact on the antioxidant capacity of nalewka prepared from walnut (S4), where the antioxidant capacity first increased from 11890 μM TE/100 mL to 16253 μM TE/100 mL after 187 days, and then decreased to 12041 (Table 3) after 439 days. The significant increase in the antioxidant capacity of walnut nalewka (S1) in the initial stage of aging can be caused by the continuous presence of walnut fruit in the nalewka, which contributes to the continuous maceration process. However, excessive extending of the fruit maceration time leads to degradation or oxidation of phenolic compounds^4^.

Generally for other tinctures, except of walnut nalewka (S4) and bilberry nalewka (S8) (Table 3), the observed effect of aging on the antioxidant capacity is not so significant. Virtually in all cases except of bilberry nalewka (S8) (Table 3), a slight increase in antioxidant capacity was observed at the first stage of aging, followed by a decrease below the initial value. The dominant class of phenolic compounds in the investigated fruits are anthocyanins. According to the literature data anthocyanin dyes are the least stable components of nalewkas, because their durability is impacted by multiple factors such as chemical structure, concentration, pH, light, presence of copigments, oxygen, sugars and products of sugar degradation, as well as storage time and temperature. They are readily oxidized in the presence of molecular oxygen, especially in the presence of quinones. Quinones, which are highly reactive molecules, are subjected to condensation reactions that result in dark-colored polymers, with structures yet to be fully understood^40^. Typically, lowering the anthocyanin content in food preserves reduces the attractiveness of the product to the consumer, but for some preserves the change to reddish color is acceptable, e.g. dried fruit or black tea. It seems that for nalewkas, traditionally perceived as long-lasting products, it is acceptable for the shade of color to shift towards brown. Nalewkas are often made from herbs and spices or with their addition, which usually results in products that are free from anthocyanins and brown in color^40^. Furthermore, on fruit products, it has been shown that the content of ascorbic acid in fruits used for production of juices or nalewkas has a very significant effect on the antioxidant capacity, as well as on the polyphenol content during aging, in addition to the above mentioned factors^41^. Fruits containing more ascorbic acid, used in production of preserves, resulted in a higher decrease of anthocyanins during the aging process in the tested food products due to faster decomposition of anthocyanins, compared with fruits containing less ascorbic acid, which is associated with a decrease in the antioxidant capacity. Additionally, a presence in the environment of tartaric acid or fructose also accelerated the degradation of anthocyanins^41^. Depending on the type of nalewka, after 93 days till about 196 days of aging there was a slight increase in the antioxidant capacity, later on the value of antioxidant capacity decreased (Table 3). The decline in the value of the antioxidant capacity after a certain aging time for nalewkas made from fruit may be due to the degradation of anthocyanins resulting from the presence of ascorbic acid in the fruits they are made from.

In case of bilberry nalewka (S8), an increase of the antioxidant capacity was observed in the first 70 days, followed by fluctuations in the antioxidant capacity within the margin of error for a further 81 days, followed by an intensive increase in the antioxidant capacity after 390 days of aging (Table 3). The change in the antioxidant capacity for the nalewka S8 is different from all the others. Bilberry nalewka (S8) is very turbid due to the presence of fruit residue in the form of pulp. The preparation process of this nalewka does not include filtration and the resulting suspension most likely causes a further maceration process, similar to that in case of the walnut nalewka.

In summary, based on the conducted study on the effects of aging time on the antioxidant capacity of nalewkas, it can be concluded that the aging time is a characteristic parameter for a given nalewka. It depends on how the nalewka is produced, as well as the type of fruit it was made from. Virtually for every nalewka (except of S8), extended aging time resulted in a decrease in the antioxidant capacity, most likely due to degradation or oxidation of phenolic compounds caused by various factors.

## Conclusions

The results of our studies show that all homemade nalewkas exhibit strong antioxidant properties and are characterized by very high TEAC_DPPH_ values.

Homemade green walnuts nalewka has the highest antioxidant capacity TEAC_DPPH_ value not only for alcoholic beverages, but also among food products.

Moreover, the impact of the production method and fruit type used on the TEAC_DPPH_ value was noted. Homemade nalewkas have much better antioxidant properties than their commercially produced counterparts.

On the basis of statistical analysis of the obtained data it could be concluded that the factors contributing to the nalewkas’ antioxidant power are total phenolic content determined by the FC method and the aromatic proton content, determined by NMR spectroscopy.

Changes in antioxidant capacity over the time depending on the production method and the type of fruit used to obtain each nalewka.
